# Treatment of infantile neuroaxonal dystrophy with RT001: A di‐deuterated ethyl ester of linoleic acid: Report of two cases

**DOI:** 10.1002/jmd2.12116

**Published:** 2020-03-27

**Authors:** Darius Adams, Mark Midei, Jahannaz Dastgir, Christina Flora, Robert J Molinari, Frederic Heerinckx, Sarah Endemann, Paldeep Atwal, Peter Milner, Mikhail S. Shchepinov

**Affiliations:** ^1^ Atlantic Health System Goryeb Children's Hospital Morristown New Jersey USA; ^2^ Clinical Division Retrotope, Inc. Los Altos California USA

**Keywords:** INAD, NBIA, neurodegeneration, PLA2G6, PLAN

## Abstract

**Background:**

Infantile neuroaxonal dystrophy (INAD) is a rare, autosomal recessive disease due to defects in *PLA2G6* and is associated with lipid peroxidation. RT001 is a di‐deuterated form of linoleic acid that protects lipids from oxidative damage.

**Methods:**

We evaluated the pharmacokinetics (PK), safety, and effectiveness of RT001 in two subjects with INAD (subject 1: 34 months; subject 2: 10 months). After screening and baseline evaluations, subjects received 1.8 g of RT001 BD. PK analysis and clinical evaluations were made periodically.

**Main findings:**

Plasma levels of deuterated linoleic acid (D2‐LA), deuterated arachidonic acid (D2‐AA), D2‐LA to total LA, and D2‐AA to total AA ratios were measured. The targeted plasma D2‐LA ratio (>20%) was achieved by month 1 and maintained throughout the study. RBC AA‐ratios were 0.11 and 0.18 at 6 months for subjects 1 and 2; respectively. No treatment‐related adverse events occurred. Limited slowing of disease progression and some return of lost developmental milestones were seen.

**Conclusions:**

Oral RT001 was administered safely in two subjects with INAD. Early findings suggest that the compound was well tolerated, metabolized and incorporated in the RBC membrane. A clinical trial is underway to assess efficacy.

**SYNOPSIS**

This article describes the first use of RT001 to treat two infants with INAD.

## 1 INTRODUCTION

INAD is a rare inherited neurological disorder (MIM 256600).^1^ It usually becomes apparent after age 6 months with slowing of motor and cognitive development and regression of previously acquired skills.^2^ The disease onset may vary according to the underlying genetic defect. Once it begins, however, the disease is relentlessly progressive, and death usually occurs between 5 and 10 years, often from loss of bulbar function leading to aspiration pneumonia.^3^, ^4^, ^5^ No drugs or therapies exist that alter the course of the disease.

INAD is an autosomal recessive disorder due to pathological variations in both copies of the *PLA2G6* gene (chromosome 22q13.1) which encodes a phospholipase (iPLA2β [MIM 603604]).^4^, ^6^, ^7^ This enzyme performs a critical housekeeping function in all cell membranes, where polyunsaturated fatty acids (PUFAs) are prone to lipid peroxidation (LPO).^8^ Membrane repair is most critical in conditions of high oxidative stress, as found in the mitochondria of highly metabolic tissues. The loss of iPLA2β function is associated with functional and structural abnormalities; this loss of function is most notable in neurons, where the damaged membranes accumulate as spheroids leading to early cell death and neurodegeneration.^9^, ^10^ Mutations in *PLA2G6* have been associated with a variety of neurodegenerative conditions including INAD, atypical NAD, and young‐onset Parkinson's disease. These diseases vary according to the degree of iPLA2β impairment. INAD represents the expression of the most severe form of *PLA2G6* variation with elevated mitochondrial lipid peroxidation and mitochondrial dysfunction.^9^

RT001 is a deuterated homologue of linoleic acid that makes membrane PUFAs resistant to LPO. RT001 has proven beneficial in a recent *Drosophila* INAD model.^9^ Reduced LPO, after treatment with RT001, was associated with recovery of the mitochondrial membrane potential of *PLA2G6* mutant human fibroblasts and with rescue of the locomotor deficits in iPLA2‐VIA knockout flies.^9^ The protective effects of deuterated PUFAs is nonlinear. Inhibition of LPO occurs even when the deuterated compounds are present at relatively low levels. A strong protective effect of deuterated linoleic acid (D2‐LA) against LPO is seen when a threshold of 20% exists in liposomal lipid bilayers.^11^ Treatment with RT001 has shown early signs of efficacy in patients with Friedreich's ataxia, a disorder of intracellular free‐iron imbalance that initiates LPO, resulting in increased oxidative stress and mitochondrial dysfunction.^12^ RT001 has been used in 64 subjects in clinical trials and has been well tolerated. In these clinical trials, a single, self‐limited episode of steatorrhea was categorized as a serious adverse event that was drug‐related. Here, we report the safety, pharmacokinetic (PK) and clinical functional outcome measures of RT001 in two INAD patients enrolled in complementary Expanded Access protocols.

## 2 MATERIALS AND METHODS

All procedures followed were in accordance with the ethical standards of the responsible committee on human experimentation (institutional and national) and with the Helsinki Declaration of 1975, as revised in 2000. Informed consent was obtained from all patients for being included in the study. The protocols were developed independently at the two sites and approved by their respective institutional review boards. Two subjects with classical INAD signs, both with two mutant *PLA2G6* alleles, were examined with a video‐recorded, structured, neurological examinations at baseline, and 6 months after the initiation of treatment with RT001 1.8 g po BID. Subject 1 also underwent evaluation at 1 year. Between clinical visits, parents completed a version of the MPS‐Health Assessment Questionnaire adapted for INAD patients.

Because no accepted tool for assessing the severity of INAD exists, we sought to develop a scale to assess patients with INAD. Other scales most commonly employed (eg, CHOP‐INTEND, Modified Ashworth, Hammersmith Functional Motor Scale, etc.) do not accurately gauge current severity of INAD nor are they sensitive/specific enough to monitor disease progression. After meeting with international leaders in the diagnosis and treatment of INAD, we produced a scale that includes five main categories of pediatric developmental evaluation: (a) gross motor‐and‐truncal‐stability skills, (b) fine motor skills, (c) bulbar function, (d) ocular function, and (e) temporo‐frontal function, and a functional evaluation of the autonomic nervous system. In a prospective natural history study of INAD patients, a significant correlation between the total neurological assessment score and months since symptom onset was demonstrated, with a statistically significant (*P* < .005) correlation between assessment score and disease onset (Atwal et al, personal communication^13^).

This INAD rating scale was developed to evaluate the video‐recorded neurological examinations by a single physician remotely. This scale included multiple developmental elements grouped according to the following categories: gross motor, fine motor, bulbar, ocular, and temporofrontal. A complete listing of individual components in the novel rating scale listed in Data S1. Each element in the scale is assigned a score of 0, 1, or 2, according to predefined scores. In general, 0 was assigned for an absent developmental function, 1 for an intermediate, and 2 for an advanced function. Currently, the INAD rating scale is a novel tool that is not yet validated. The utility of this tool is being validated as part of ongoing expanded access, treatment, and natural history studies of INAD patients.

Subjects were evaluated periodically with PK analysis. Plasma concentrations of deuterated (D2) and nondeuterated LA and AA were measured. These compounds were also in the RBC membranes as an indicator of membrane incorporation. The plasma and RBC membrane ratios were calculated by dividing the deuterated compound concentration by the sum of the deuterated and nondeuterated concentrations. A detailed description of the analytical methods is provided in Data S2.

## 3 RESULTS

### 3.1 Demographic and genetic characteristics

Both subjects had commercial genetic testing performed in a CLIA/CAP certified US laboratory by way of large‐scale massively parallel sequencing (next generation sequencing [NGS]) on a TRIO exome platform. Subject 1 is a female with bi‐allelic homozygous *PLA2G6* pathogenic variants denoted c.208C>T, p.R70X, age 3 at enrollment, and remains under treatment after 34 months. Subject 2 is a male with bi‐allelic *PLA2G6* pathogenic variants denoted c.404 T>C, p.F135S (paternal) and an intragenic deletion of exon 6 (maternal), confirmed in trans by parental analysis, age 5 at enrollment, and discontinued treatment after 10 months.

### 3.2 Safety assessments

Overall, RT001 was well tolerated. Subject 1 had a brief bout of abdominal cramping after 12 months of therapy that was attributed to another treatment. A reduction in RT001 dose, followed by resumption of full dosing without issue, was done as a precaution. Subject 2 underwent gastric tube placement after 6 months of therapy to simplify feeding. Approximately 4 months later, RT001 was discontinued due to parental perception of stagnation after the initial clinical improvements, and due to their desire to restore eligibility for enrollment in other clinical trials. No serious adverse events were seen in either subject.

### 3.3 PK assessments

**Figure 1:**
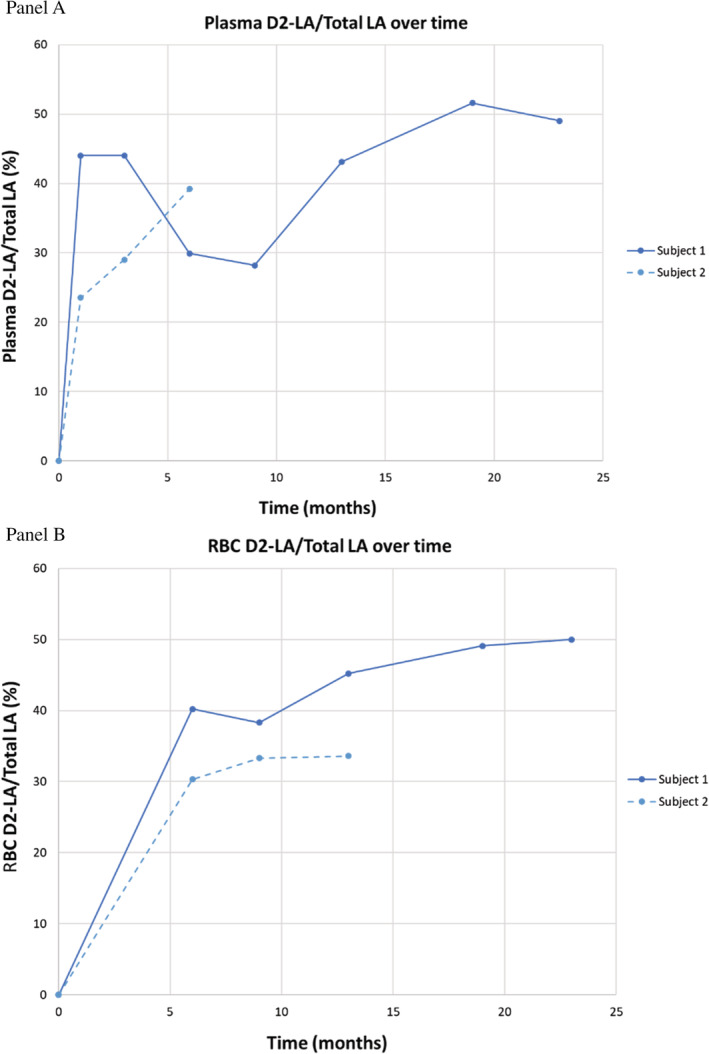
Plasma and RBC pharmacokinetic data for the two subjects is depicted graphically over time. Panel A, shows the ratio of plasma D2‐LA to total LA; Panel B, shows the ratio of RBC D2‐LA to total LA for the two subjects

**Figure 2:**
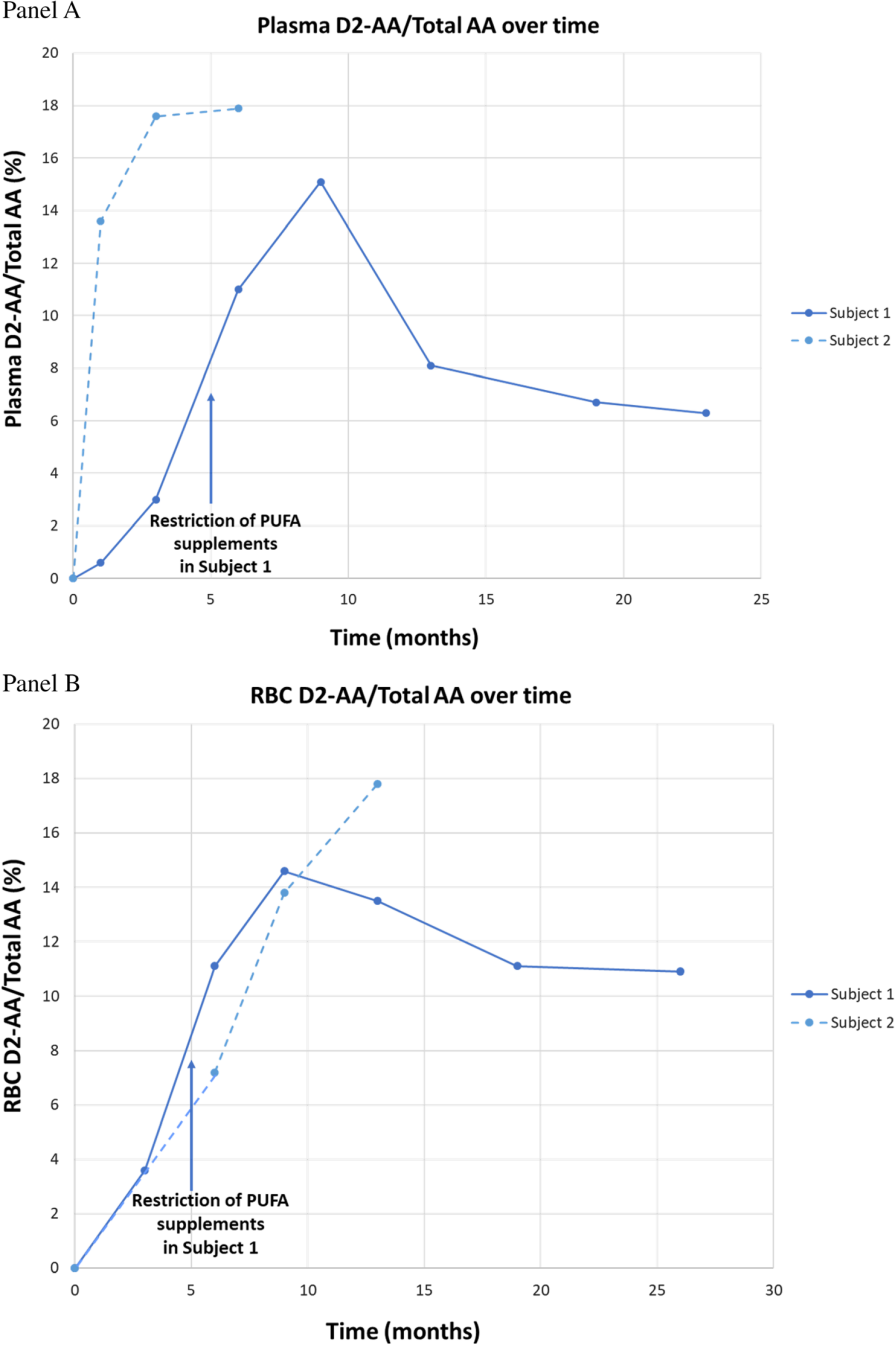
Plasma and RBC pharmacokinetic data for the two subjects is depicted graphically over time. Panel A, shows the ratio of plasma D2‐AA to total AA; Panel B shows the ratio of RBC D2‐AA to total AA for the two subjects

Plasma D2‐LA was present in significant quantities within 1 month of starting RT001 in both subjects. Figure 1 shows the ratio of D2‐LA to total LA in the plasma (Panel A) and the RBC membranes (Panel B). These values exceeded 20% within the first month for both subjects and continued to increase throughout RT001 exposure. Elongation of D2‐LA into D2‐AA occurred, although the concentration of plasma D2‐AA and RBC D2‐AA was lower than for D2‐LA (Figure 2). This phenomenon was most notable in subject 1, who was receiving high levels of supplementary PUFAs. These included supplements of capsules containing omega 3 and omega 6 PUFAs as well as dietary chia seeds and almond butter. The capsules and dietary supplements were discontinued ~5 months after the initiation of RT001 therapy. After dietary modification to limit PUFAs, D‐AA levels increased in proportion to those seen in subject 2.

## 4 FUNCTIONAL ASSESSMENTS

**Figure 3:**
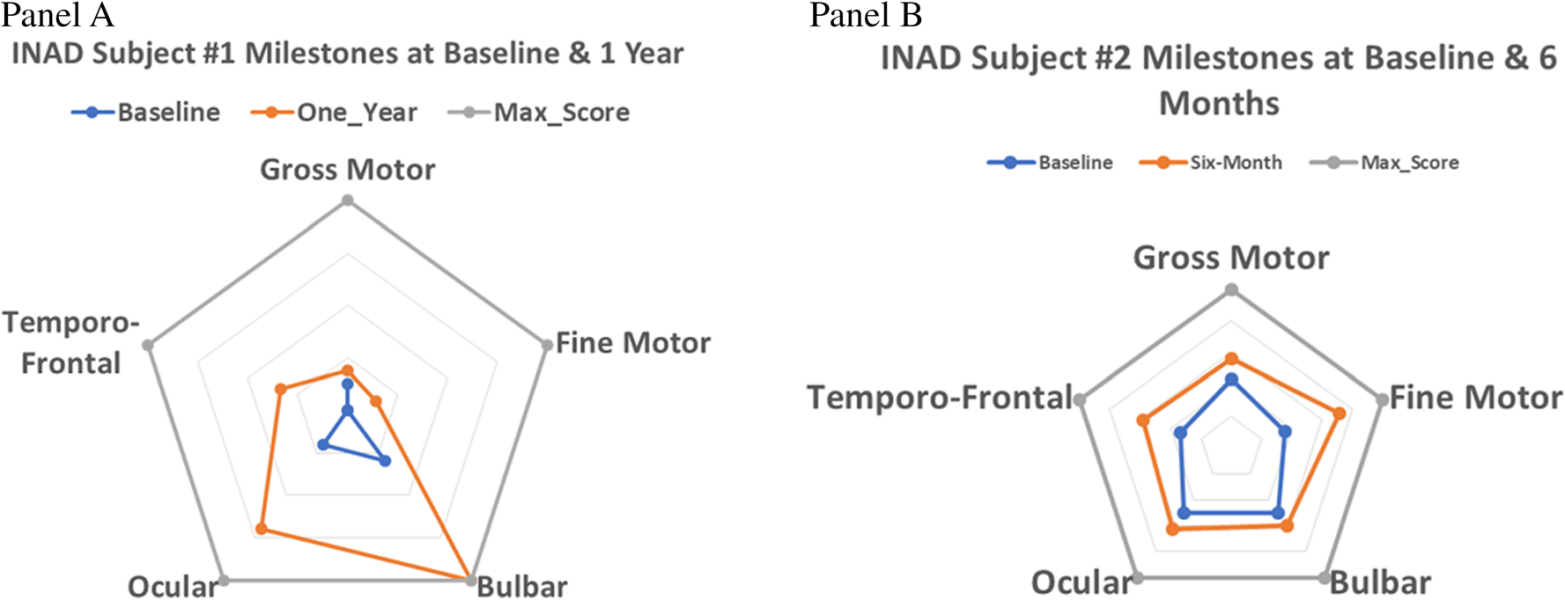
Radar plots of the milestone category scoring for the two subjects. Panel A, depicts Subject #1 at maximal follow‐up of 1 year, and Panel B depicts Subject 2 at maximal follow‐up of 6 months

Both subjects have shown improvement over baseline for INAD rating score (Subject 1: 7‐26; Subject 2: 27‐39; max 62), and in the number of elements showing improvement (Subject 1: 16; Subject 2: 12; max 31). Both subjects showed improvement in at least one component of each developmental category. Radar plots of the milestone categories comparing baseline scoring to scoring at follow‐up for the two subjects are shown in Figure 3. Although these results appear to be promising, they remain too preliminary to derive any conclusions as the rating scale has not yet been validated, and more studies are underway to assess its utility.

### 4.1 Subject 1 narrative

Improved ability to hold her head upright and grasping of small objects including food and a spoon were noted within 6 months of treatment initiation. Improved bulbar function with swallowing of saliva and solid food was observed at 12 months. Tracking of human faces, smiling and overall interaction with parents (milestones previously lost) were also observed after 12 months of treatment. Severe constipation was improved significantly with treatment. The subject remains on treatment after 34 months; although the re‐gaining of additional lost milestones has not occurred, the early observed improvements have not reversed.

### 4.2 Subject 2 narrative

Improvements in alertness, participation, vocalization, and fine motor control were observed after 1 month of treatment with RT001. The subject regained the ability to initiate steps with walker assistance and to imitate a song. After 3 months, spontaneous reaching for objects returned, and eight steps were taken in a walker. The subject was observed to have improved engagement and no regression in skills. After 6 months on RT001, parents reported that cognitive interaction and attention was improved, with no regression in gross motor skills. Throughout the study period, the subject did not see improvement in oromotor skills, and a gastrostomy tube was placed while on RT001 in the setting of ongoing feeding difficulty. The parents reported that the subject began to have decline in motor skills, including worsening dystonic posturing, after 6 months and while on RT001. No formal assessments were completed after 6‐month assessment. The subject's parents withdrew from the study after 10 months due to parental perception of stagnation after the initial clinical improvements, and due to their desire to restore eligibility for enrollment in other clinical trials.

## 5 DISCUSSION

In this report of two subjects with INAD, oral RT001 was administered orally and was well‐tolerated. D2‐LA was present in the plasma of both subjects within 1 month of RT001 initiation. Elongation to D2‐AA was also demonstrated with significant levels measured in the plasma. Both compounds were also incorporated at significant levels into the RBC membranes. No serious adverse events clearly related to study drug occurred in either subject.

The key step in lipid peroxidation of the mitochondrial membrane is hydrogen abstraction from a bis‐allylic site.^14^ Deuteration of these bis‐allylic sites slows peroxidation and protects against further oxidative damage and formation of toxic by‐products.^15^ In preclinical models of IND enabling toxicity, we observed efficient conversion of RT001 into D2‐AA and expected levels of incorporation of the drug metabolite in each tissue type—unchanged from AA levels in animals dosed with normal fats.^16^ The current study demonstrates that RT001 is absorbed and equilibrates with plasma and cellular levels of both D2‐LA and D2‐AA in patients with INAD.

The presence of oxidative stress in INAD has been well documented in cell and animal models.^9^, ^10^ *PLA2G6* function is required for repair of peroxidized lipids for membrane homeostasis in normal cells. Its absence is associated with accumulation of peroxidized lipid byproducts, mitochondrial dysfunction, and early cell death. The high concentration of PUFAs in the inner mitochondrial membrane makes it especially vulnerable to lipid peroxidation. The demonstration D2‐LA and D2‐AA incorporation into RBC membranes as a surrogate for other cellular and mitochondrial membranes may be responsible for some of the clinical observations in this report of two cases. Further analysis of efficacy in the more expansive open‐label clinical study currently underway is warranted.

Within 1 month of dosing and continuing through 34 and 6 months, respectively, both subjects with INAD demonstrated no further loss of additional milestones. This effect was sustained up to 34 months after RT001 initiation in subject 1. In a disease marked by inexorable decline and milestone loss, stabilization of regression without further losses of milestones appears promising, and warrants further study. Parents and caregivers reported that subject 2 experienced a stabilization in his clinical course, but no additional milestones returned after this early improvement. In addition, bulbar function never changed, and this was a particularly disturbing feature of his disease process. Ultimately, his parents opted to discontinue therapy after 10 months.

Despite some of these encouraging observations of the effect of RT001 in INAD, it is too early to derive any conclusions without more studies to assess the efficacy of this drug. Parallel open‐label treatment (n = 19; NCT03999814) and prospective natural history (n = 40; NCT04027816) studies are currently underway and may provide more definitive answers.

Even in our small sample of two subjects, the pathogenic variant spectrum observed in our patients included nonsense (R70X), missense (F135S) and a deletion of exon 6. To our knowledge, a specific functional region affected by this deletion has not been defined; all enzymatic function may be affected including ATP‐dependent protein binding, calcium‐independent phospholipase A2 activity, calmodulin binding, and hydrolase activity. The perceived discrepancy in clinical response may be related to differences in the severity of the gene variants, subject age, parental expectations, and a variety of other factors. A multi‐center, 19‐subject open‐label study of RT001 in INAD is currently underway that should provide greater objectivity in the analysis. The developmental observations made in these expanded access protocols will serve as the basis for the novel rating scale that will be used as the primary endpoint in a prospective natural history study and a parallel treatment protocol.

## CONFLICT OF INTEREST

D. A., J. D., and C. F. declare no conflict of interest. P. M., F. H., M. M., P. A., and M. S., are employed by and holds stock in Retrotope. R. M. is a founder and holds stock in Retrotope, Inc. Sarah Endemann is employed by Retrotope. All authors confirm that the content of the article has not been influenced by the sponsor.

## AUTHOR CONTRIBUTIONS

Darius Adams: principal investigator at the site, conducted the study, analyzed data, and assisted in manuscript writing. Mark Midei: analyzed data, served as medical monitor, wrote the manuscript. Jahannaz Dastgir: Served as investigator, conducted the study at the site. Christina Flora, LGC: Provided genetic counseling, conducted the study at the site, collected data, analyzed data. Robert J Molinari: Designed the study, analyzed data, and edited the manuscript. Frederic Heerinckx: Designed the study, conducted the study, and analyzed the PK data. Sarah Endemann: Conducted the study, collected PK data, and managed relationships with subjects and families. Paldeep Atwal: analyzed genetic data, edited manuscript. Peter Milner: Designed the study, analyzed the data, and edited manuscript. Mikhail S. Shchepinov: Designed the study, invented the drug, and edited the manuscript.

## ETHICS STATEMENT

These studies were conducted in accordance with the U.S. Food and Drug Administration Code of Federal Regulations (CFR), 21 CFR Part 312.20, as well as the Declaration of Helsinki (2013) and the International Conference on Harmonization Guidelines for Good Clinical Practice (1997). The protocols were developed independently at the two sites and approved by their respective institutional review boards (Goryeb Children's Hospital and Duke University).

## A PATIENT CONSENT STATEMENT

All procedures followed were in accordance with the ethical standards of the responsible committee on human experimentation (institutional and national) and with the Helsinki Declaration of 1975, as revised in 2000 (5). Informed consent was obtained from both patients for being included in the study. Proof that informed consent was obtained is available upon request.

## Supporting information


**Data** **S1**: Supporting Information

## References

[1] Aicardi J , Castelein P . Infantile neuroaxonal dystrophy. Brain. 1979;102:727‐748. 509195 10.1093/brain/102.4.727

[2] Nardocci N , Zorzi G , Farina L , et al. Infantile neuroaxonal dystrophy: clinical spectrum and diagnostic criteria. Neurology. 1999;52:1472‐1478. 10227637 10.1212/wnl.52.7.1472

[3] Kurian MA , Morgan NV , MacPherson L , et al. Phenotypic spectrum of neurodegeneration associated with mutations in the PLA2G6 gene (PLAN). Neurology. 2008;70:1623‐1629. 18443314 10.1212/01.wnl.0000310986.48286.8e

[4] Kurian MA , McNeill A , Lin JP , et al. Childhood disorders of neurodegeneration with brain iron accumulation. Dev Med Child Neurol. 2011;53:394‐404. 21480873 10.1111/j.1469-8749.2011.03955.x

[5] Iodice A , Spagnoli C , Salerno GG , et al. Infantile neuroaxonal dystrophy and PLA2G6‐ associated neurodegeneration: an update for the diagnosis. Brain Dev. 2017;39:93‐100. 27884548 10.1016/j.braindev.2016.08.012

[6] Morgan NV , Westaway SK , Morton JE , et al. PLA2G6, encoding a phospholipase A2, is mutated in neurodegenerative disorders with high brain iron. Nat Genet. 2006;38:752‐754. 16783378 10.1038/ng1826 PMC2117328

[7] Salih MA , Mundwiller E , Khan AO , et al. New findings in a global approach to dissect the whole phenotype of PLA2G6 gene mutations. PLoS ONE. 2013;8:e76831. 24130795 10.1371/journal.pone.0076831 PMC3792983

[8] Balsinde J , Balboa MA . Cellular regulation and proposed biological functions of group VIA calcium‐independent PLA2 in activated cells. Cell Signal. 2005;17:1052‐1062. 15993747 10.1016/j.cellsig.2005.03.002

[9] Kinghorn KJ , Castillo‐Quan JI , Bartolome F , et al. Loss of PLA2G6 leads to elevated mitochondrial lipid peroxidation and mitochondrial dysfunction. Brain. 2015;138:1801‐1816. 26001724 10.1093/brain/awv132 PMC4559908

[10] Beck G , Sugiura Y , Shinzawa K , et al. Neuroaxonal dystrophy in calcium‐independent phospholipase A2b deficiency results from insufficient remodeling and degeneration of mitochondrial and presynaptic membranes. J Neurosci. 2011;31:11411‐11420. 21813701 10.1523/JNEUROSCI.0345-11.2011 PMC6623367

[11] Firsov AM , Fomich MA , Bekish AV , et al. Threshold protective effect of deuterated polyunsaturated fatty acids on peroxidation of lipid bilayers. FEBS J. 2019 Jun;286(11):2099‐2117. 30851224 10.1111/febs.14807

[12] Zesiewicz T , Heerinckx F , DeJager R , et al. Randomized, clinical trial of RT001: early signals of efficacy in Friedreich's ataxia. Mov Disord. 2018;33:1000‐1005. 29624723 10.1002/mds.27353

[13] Atwal PS , Heerinckx F , Midei M , Milner P . A neurological scale for infantile neuroaxonal dystrophy. Neurology 2020:94(15):11–009. 10.1186/s13023-020-01479-5 PMC7392694 32727524

[14] Porter NA , Caldwell SE , Mills KA . Mechanisms of free radical oxidation of unsaturated lipids. Lipids. 1995;30:277‐290. 7609594 10.1007/BF02536034

[15] Hill S , Lamberson CR , Xu L , et al. Small amounts of isotope‐reinforced polyunsaturated fatty acids suppress lipid autoxidation. Free Rad Biol Med. 2012;53:893‐906. 22705367 10.1016/j.freeradbiomed.2012.06.004 PMC3437768

[16] Shchepinov MS , Roginsky VA , Brenna JT , et al. Deuterium protection of polyunsaturated fatty acids against lipid peroxidation: a novel approach to mitigating mitochondrial neurological diseases In: Watson RS, De Meester F, eds. In Omega 3 Fatty Acids in Brain and Neurological Health. Amsterdam: Elsevier Academic Press; 2014:373‐383.

