# Supplementary material for: Treatment of infantile neuroaxonal dystrophy with RT001: A di‐deuterated ethyl ester of linoleic acid: Report of two cases
Source: JIMD Rep. 2020 Mar 27;54(1):54–60. doi: 10.1002/jmd2.12116 (PMC7358664; doi:10.1002/jmd2.12116)
Supplement: Supplementary file 1 — Data S1: Supporting Information [file JMD2-54-54-s001.docx]

**Supplement 1. The novel rating scale used to evaluate the subjects over time included 5 developmental categories of affected in INAD. Each category included multiple elements for which a score of 0-2 was assigned.**

| **Category** | **Milestone** |
| --- | --- |
|  |  |
| **Gross Motor** | Hold head upright |
|  | Tone of trunk/core |
|  | Roll over |
|  | Sit |
|  | Stand aided or unaided |
|  | Axial strength |
|  | Hand grip and pes cavus or pes equinus |
|  | Walk aided or unaided |
| **Fine Motor** | Reaches for objects or bell |
|  | Picks up food or spoon |
|  | Grasps small objects |
|  | Rings bell |
|  | Transfers objects |
|  | Place one block on another |
|  | Point to objects in book |
| **Bulbar** | Swallows saliva |
|  | Swallows pureed food |
|  | Swallows solid food |
|  | Bite strength |
|  | Nourishes by syringe or feeding tube |
| **Ocular** | Nystagmus |
|  | Strabismus |
|  | Tracks human face |
|  | Tracks object |
|  | Optic atrophy/temporal pallor |
| **Temporo-frontal** | Interacts with parents or examiner |
|  | Responds to verbal commands |
|  | Repeats simple sounds |
|  | Smiles |
|  | Speaks individual words |
|  | Puts words together |

**Supplement 2. Analytical and Pharmacokinetic Methods**

Plasma concentrations of RT001 (combined free and ester forms) and non-deuterated analogs were determined by LC-MS. Blood was collected in K_2_EDTA anticoagulant tubes, centrifuged to obtain plasma and red blood cells (RBCs), and frozen until analyzed. The samples were hydrolyzed to free fatty acids and analyzed by high performance LC-MSby GLP-validated methods. Low-intensity D2 and high- intensity H_2_ signals were measured with high accuracy at Ricerca Biosciences, using an AB SCIEX 6500 tandem-quadrupole MS. The ^13^C_2_ isotopomer signal was used as a calibration standard for RT001; a poly-deuterated internal standard was employed. The enzymatic elongation/desaturation metabolite, 13,13- D2-Ara [10], was also measured, in plasma and RBC.

The study was conducted in accordance with the U.S. Food and Drug Administration Code of Federal Regulations (CFR), 21 CFR Part 312.20, as well as the Declaration of Helsinki (2013) and the International Conference on Harmonization Guidelines for Good Clinical Practice (1997) [5,6]. The protocol was reviewed and approved by institutional review boards at the respective sites.
